# Pediatric Obesity and Vitamin D Deficiency: A Proteomic Approach Identifies Multimeric Adiponectin as a Key Link between These Conditions

**DOI:** 10.1371/journal.pone.0083685

**Published:** 2014-01-03

**Authors:** Gillian E. Walker, Roberta Ricotti, Marta Roccio, Stefania Moia, Simonetta Bellone, Flavia Prodam, Gianni Bona

**Affiliations:** 1 Laboratory of Clinical Pediatrics, Department of Health Sciences, Università del Piemonte Orientale “Amedeo Avogadro”, Novara, Italy; 2 Division of Pediatrics, Department of Health Sciences, Università del Piemonte Orientale “Amedeo Avogadro”, Novara, Italy; Instituto de Ciencia de Materiales de Madrid - Instituto de Biomedicina de Valencia, Spain

## Abstract

Key circulating molecules that link vitamin D (VD) to pediatric obesity and its co-morbidities remain unclear. Using a proteomic approach, our objective was to identify key molecules in obese children dichotomized according to 25OH-vitamin D (25OHD) levels. A total of 42 obese children (M/F = 18/24) were divided according to their 25OHD3 levels into 25OHD3 deficient (VDD; n = 18; 25OHD<15 ng/ml) or normal subjects (NVD; n = 24; >30 ng/ml). Plasma proteomic analyses by two dimensional (2D)-electrophoresis were performed at baseline in all subjects. VDD subjects underwent a 12mo treatment with 3000 IU vitamin D3 once a week to confirm the proteomic analyses. The proteomic analyses identified 53 “spots” that differed between VDD and NVD (p<0.05), amongst which adiponectin was identified. Adiponectin was selected for confirmational studies due to its tight association with obesity and diabetes mellitus. Western Immunoblot (WIB) analyses of 2D-gels demonstrated a downregulation of adiponectin in VDD subjects, which was confirmed in the plasma from VDD with respect to NVD subjects (p<0.035) and increased following 12mo vitamin D3 supplementation in VDD subjects (p<0.02). High molecular weight (HMW) adiponectin, a surrogate indicator of insulin sensitivity, was significantly lower in VDD subjects (p<0.02) and improved with vitamin D3 supplementation (p<0.042). A direct effect *in vitro* of 1α,25-(OH)2D3 on adipocyte adiponectin synthesis was demonstrated, with adiponectin and its multimeric forms upregulated, even at low pharmacological doses (10^−9^ M) of 1α,25-(OH)2D3. This upregulation was paralleled by the adiponectin interactive protein, DsbA-L, suggesting that the VD regulation of adiponectin involves post-transciptional events. Using a proteomic approach, multimeric adiponectin has been identified as a key plasma protein that links VDD to pediatric obesity.

## Introduction

As with obesity, vitamin D (VD) deficiency is reaching epidemic proportions worldwide, in both pediatric as well as adult populations [1]. Evidence is accumulating to suggest that there is a potential link between obesity and VD deficiency among global populations [2], [3]. Beyond vitamin D's historic role in bone mineralization to its more recent association with allergy development [1], [4], reports to date have linked VD deficiency to hypertension, diabetes mellitus and insulin resistance (IR), non-alcoholic fatty liver disease (NAFLD) and the metabolic syndrome [1], [3], [5]–[11]. Consequently, VD deficiency may no longer be a condition but rather a mediator of metabolic diseases responsible for the long-term health outcomes of obese children.

Vitamin D is a group of fat soluble prohormones, with the two major forms being ergocalciferol (VD2) and cholecalciferol (VD3)[2], [3]. *In vivo*, VD3 and VD2 are metabolized by the liver to produce 25-dihydroxyvitamin D3 (25-OHD3) or 25-OHD2. These metabolites are then further metabolized by the kidney to produce the bioactive forms 1α,25-(OH)2D3 and 1α,25-(OH)2D2. The bioactive form of VD3, 1α25-dihydroxyvitamin D3 (1α,25-(OH)2D3), functions as a pleiotropic hormone controlling gene expression in numerous cell types and tissues regulating proliferation, differentiation and cell survival [1]. These activities are achieved principally via the cytosolic/nuclear vitamin D receptor (VDR) signal-transduction pathways and VD responsive elements (VDRE) found on numerous key genes, with rapid responses occurring via VDR localized in the plasma and endoplasmic reticulum (ER) membranes [12]. The VDR has been found in more than 40 tissues including pancreatic beta-cells, smooth muscle cells, monocytes and adipocytes [13]. As such, it is hypothesized that VD deficiency could lead to complex disease phenotypes, including obesity.

Excess body fat is associated with an increased risk of suboptimal VD status [3], [14]. It is, however, unclear as to whether poor VD status is a consequence of obesity or is actively involved in its development [1]–[3]. Until now, data regarding the role of VD are inconclusive as the majority of results are derived from association studies, with intervention and longitudinal studies lacking [15]. Cross-sectional studies have principally focused on classical cardiovascular risk factors such as blood pressure, fasting glucose and lipids in both adults and children [11], [16], with studies in pediatric subjects revealing that different VD cut-offs relate to specific cardiovascular outcomes [11], [17]. While respecting that a role exists, the links between VD and obesity remain to be elucidated.

In the search for biomarkers that are representative of individual disease states, proteomic analyses can evaluate globally expressed and activated protein pathways in both physiological and pathological conditions. A proteomic approach can differentiate phenotypes of diseases as well as investigate mechanisms for target therapies. Recently, a proteomic study conducted in adult obesity revealed that VD binding globulin could be a marker of changes in body fat mass [18]. As such, to investigate a functional relationship between VD status and pediatric obesity, the aim of this study was to firstly using a proteomic approach, target potential plasmatic biomarkers that could link VD deficiency to pediatric obesity. The second aim was to then verify that such markers could be modulated by VD supplementation *in vivo*, and finally to confirm such effects *in vitro* and shed light as to the biological mechanisms involved in the direct effect of 1α,25-(OH)2D3.

## Subjects and Methods

### Subjects

In this study, we recruited consecutively 97 children and adolescents aged between 5–18 yrs referred to the Pediatric Endocrine Service of the “Ospedale Maggiore della Carità” in relation to obesity from October 2009. Subjects were eligible if they were healthy, had a body mass index (BMI) that exceeded the 95^th^ percentile according to the Italian growth charts [19], were diet-naïve and presented 25-OHD3 levels <15.0 ng/ml (deficiency; VDD) or >30 ng/ml (sufficiency; NVD). The level of 15.0 ng/ml 25-OHD3 was chosen as it represents the value below which cardiovascular risk factors are significantly associated to 25-OHD3 in children and adolescents, as described by the National Health and Nutrition Examination Survey [10], as well as in adults [16]. Subjects with intermediate 25-OHD3 levels (15.0–30.0 ng/ml) were excluded from the study to avoid the potential interference of VD hypovitaminosis. Exclusion criteria included the presence of diabetes mellitus, the use of pharmaceuticals which could influence glucose and lipid metabolism, blood pressure or appetite, as well as endocrine or genetic obesity, or a low birth weight.

The protocol was conducted in accordance with the declaration of Helsinki of 1975 as revised in 1983 and was approved by a Local Ethic Committee (Ethics Committee AOU “Maggiore della Carità” di Novara, ASL BI, ASL NO, ASL VC ASL VCO; protocol 199/CE; study CE 14/11; www.maggioreosp.novara.it). A written informed consent was obtained by all parents before the evaluations where the purpose of the study was carefully explained.

### Anthropometric and biochemical measurements

All the subjects underwent a clinical evaluation using the Italian growth charts [19]. Subjects which were assigned to VDD (n = 18), were evaluated at baseline and after 6–12mo. Each received 3000 IU cholecalciferol (VD3) once a week (corresponding at about 400 IU daily) according to the recommendations American Academy of Pediatrics 2008 [20].

Pubertal stages were determined by an assigned group of trained physicians, using the criteria of Marshall and Tanner [21]. Height was measured to the nearest 0.1 cm by the Harpenden stadiometer and weight with light clothing to the 0.1 kg by using a manual scale. BMI was calculated as body weight divided by squared height (kg/m^2^). BMI standard deviation score (BMISDS) was calculated with the LMS method [19]. Waist circumference (WC) was measured at the high point of the iliac crest around the abdomen and was recorded to 0.1 cm. Systolic (SBP) and diastolic (DBP) blood pressure were measured three times on the left arm after 15 min at rest in the supine position and prior to other physical evaluations, by using a standard mercury sphygmomanometer; the average was used for analyses.

After a 12 h overnight fast, morning blood samples for proteomic analyses, glucose, insulin and 25-OHD3 were obtained. All subjects underwent an OGTT (1.75 g of glucose solution per kg, maximum 75 g). Insulin resistance and sensitivity were calculated using the formula of HOMA-IR and Matsuda index, respectively. Glucose was expressed in mg/dl (1 mg/dl:0,05551 mMol/liter) and insulin in µUI/ml (1 µUI/ml = 7.175 pmol/l). All measurements were performed using standardized methods in the hospital's analysis laboratory. Vitamin D as 25OHD3 serum levels (ng/ml) were assayed by a direct competitive chemiluminescent immunoassay with a CV value of 4% (Liaison® Test 25OHD total, DiaSorin Inc, Stillwater MN-USA). Human total adiponectin (µg/ml) was measured by the method of ELISA according to the manufacturer's instructions (AdipoGen Inc, Incheon, Korea), with the intra-assay and inter-assay coefficients of variation 3.8% and 5.5%, respectively. The sensitivity of the assay was 0.0001 µg/ml. Human unacylated ghrelin (pg/ml) was also measured by ELISA (BioVendor, Brno, Czech Republic), with the intra-assay and inter-assay coefficients of variation 4.4% and 4.5%, respectively. The sensitivity of the assay was <5 pg/ml. Formulas and other assays were previously described [22].

### 2D-Electrophoresis

To prepare platelet-free plasma for 2D-electrophoresis, all samples were centrifuged at 1300 rpm, 4C for 10 min followed by a further centrifugation at 2400rcf 4°C for 15 min, with storage at –80°C. Plasma protein concentrations were determined by using the DC Protein Assay (BioRad, Hercules, CA). To reduce biological variation in the proteomic analysis, as described by Mischak *et al.*, 2010 [23], a minimum of 12 samples per group was delineated. For the 1^st^ dimension, equal volumes of plasma within the range of 50 ug of protein per analysis, were re-suspended in rehydration buffer, according to the method of de Roos et al., 2008 [24] and loaded onto a 7 cm immobilized pH gradient (IPG) 3–10 strip for an overnight (O/N) active in-gel rehydration (BioRad). Isoelectric focusing (IEF) was performed at 20°C with a Protean IEF Cell (BioRad) using a total of 10,000 V/h with a maximum of 8,000 V. For the 2^nd^-dimensional separation, the IPG strips were soaked, firstly in a reduction equilibration buffer (6 M Urea, 2%SDS, 0.375 M Tris-HCl pH8.8, 20% glycerol 2% w/v DTT), followed by an alkylation buffer (6 M Urea, 2%SDS, 0.375 M Tris-HCl pH8.8, 20% glycerol 2.5% w/v iodoacetamide; BioRad). The strips were then positioned in 10% SDS-polyacrylamide gels (SDS-PAGE) run at 200 V for 40 min. Polyacrylamide gels were fixed in 10% methanol, 7% acetic acid and resolved protein spots visualized with an O/N incubation in Sypro-Ruby fluorescent total protein stain (BioRad). All samples were evaluated in duplicate.

### Image analysis

Fluorescent images of individual gels were captured with a ChemiDoc Imager using a 615–645 nM filter (630BP30; BioRad) and analyzed using PDQuest software (version 8.0) according to the manufacturer's recommendations. Briefly, the software performs an automated detection and matching of spots from all gels, calculating individual spot “volumes” by density/area integration with Sypro-Ruby filtration and Gaussian modeling. To control for slight differences in protein loading across gels, the individual spot volumes were also normalized to the total spot volume for each gel. For each protein spot, an average value for VDD and NVD were compared and subjected to Student's t-test to determine the spots that were significantly different between the two groups. Only those spots that showed a statistically significant difference with a p<0.05, were chosen for PDQuest isoelectric point (pI) and molecular weight (MW) estimations and identification.

### Western immunoblot (WIB)

Independent of the experiments performed, all samples were size-fractionated on 10% SDS-PAGE under reducing or non-reducing (NR) conditions and electro-transferred to immuno-blot polyvinylidene difluoride (PVDF) membrane (BioRad). For both plasma and conditioned medium (CM) under NR and R conditions, membranes were incubated with monoclonal anti-adiponectin (Adipogen, Inc Incheon Korea) and detected with the appropriate horseradish peroxidase-conjugated secondary antibody (Chemicon Millipore, Temecula, CA). Likewise, whole cell lysates (WCL) were analyzed with anti-DsbA-L (Abcam, Cambridge, UK) and anti-α Tubulin (Sigma). Total protein from CM was assessed by Ponceau S staining and used for normalization (Sigma). Immunoreactive proteins were detected using enhanced chemiluminescence (Pierce Biotechnology, Rockford, IL) with image capture performed using CCD-camera linked to ChemiDoc (BioRad). Results, were quantified using QuantityOne software with values presented as arbitrary units (AU) normalized to total protein concentrations.

### 3T3-L1 cell culture and treatments

To address the direct effect of 1α,25-(OH)2D3 on adipose tissue (AT), the well characterized murine 3T3-L1 preadipocyte cell model was utilized (European Collection of Cell Cultures). The preadipocytes were grown to confluency in their maintenance medium (Dulbecco's modified Eagle's medium supplemented with 10% FBS and 1% penicillin/streptomycin; Sigma) at which the cells were induced to differentiate with the addition of 500 uM isobutylemthylxanthine (IBMX; Sigma), 25 uM dexamethasone (DEX; Sigma) and 0.5 ug/ml insulin (Sigma) for 3 d, following which the medium was changed to straight insulin containing medium for an additional 3 d. To complete differentiation, the medium was returned to maintenance medium for a further 4 d, giving a total of 10 d for the formation of adipocytes. At this time, the adipocytes were then placed into serum-free medium (SFM) with an equal volume of vehicle (ethanol), or SFM with 10^−9^ M or 10^−7^ M 1α,25-(OH)2D3 (Sigma) and left to incubate for up to 48 h, with aliquots of conditioned medium (CM) removed at 7, 24 and 48 h. Aliquots were centrifuged at 1000 rpm at 4C and stored at −20C prior to electrophoretic analyses. At the 48 h time point, WCL were prepared using RIPA buffer (20 mM HEPES pH 7.4, 150 mM NaCl, 1% Triton-X 100, 1% sodium deoxycholate, 0.1% SDS, SIGMAFAST EDTA free protease inhibitor cocktail; Sigma) with concentrations determined using the BCA Protein Assay (Pierce, Rockford, IL).

### Statistical analysis

Data are expressed as mean ± SEM. Skewed variables were logarithmically transformed before analyses when necessary. Differences between groups, treatments and *in vitro* studies were compared using Mann-Whitney U or Wilcoxon test, Student's t-test or ANCOVA with BMISDS, age and sex as covariates, where appropriate. Statistical significance was assumed for p<0.05. The statistical analyses were performed with SPSS for Windows version 17.0 (SPSS; Chicago, IL).

## Results

### Baseline evaluations

Of the 97 original subjects, 55 were excluded because they did not satisfy the inclusion criteria to do the proteomic analyses with respect to their 25-OHD3 levels (VDD: <15 ng/ml; NVD >30 ng/ml). The final dataset included 42 participants, aged between 5–18 yrs (18 M/24 F). Of these, 18 were classified VDD (range: 5.40–14.20 ng/ml), with the remainder falling into the group NVD (range: 31.20–50.0 ng/ml) without differences in seasonal distribution of the samples. Age and Tanner stages were similar between the two groups. Basal evaluations demonstrated that VDD subjects were more obese, more insulin-resistant and had higher fasting glucose and DBP, when compared to NVD. The clinical and biochemical characteristics of VDD and NVD are shown in Table 1.

**Table 1:** Basal clinical and biochemical characteristics of subjects.

	NVD (25OHD >30 ng/mL)	VDD (25OHD <15 ng/mL)
**M/F**	8/16	10/8
**PP/P**	12/12	8/10
**25OHD (ng/dl)**	37.0±1.1	11.0±0.5****
**BMI (Kg/m2)**	25.6±0.7	28.3±1.3*
**BMISDS**	1.804±0.100	2.094±0.130***
**W/H**	0.59±0.01	0.62±0.10
**SBP (mmHg)**	121.4±3.3	122.5±3.1
**DBP (mmHg)**	79.7±2.1	85.2±2.6***
**Glc0' (mg/dl)**	85.2±1.8	89.1±1.5**
**Ins0'(mUI/l)**	13.9±1.2	14.6±2.1
**HOMA**	2.9±0.2	3.2±0.5**
**Matsuda index**	4.44±0.67	3.47±0.28**
**Adiponectin (AU)**	8594.6±578.2	7189.1±383.6***

AU: arbitrary unit; BMI: body mass index; DBP: diastolic blood pressure; F: female; Glc0': fasting glucose; HOMA: homeostatic model assessment; Ins0': fasting insulin; M: male; PP: prepubertal: P: pubertal; SBP: systolic blood pressure: * p = 0.06; ** p<0.05; *** p<0.03; **** p<0.0001.

### 2D-electrophoretic analysis

**Figure 1:**
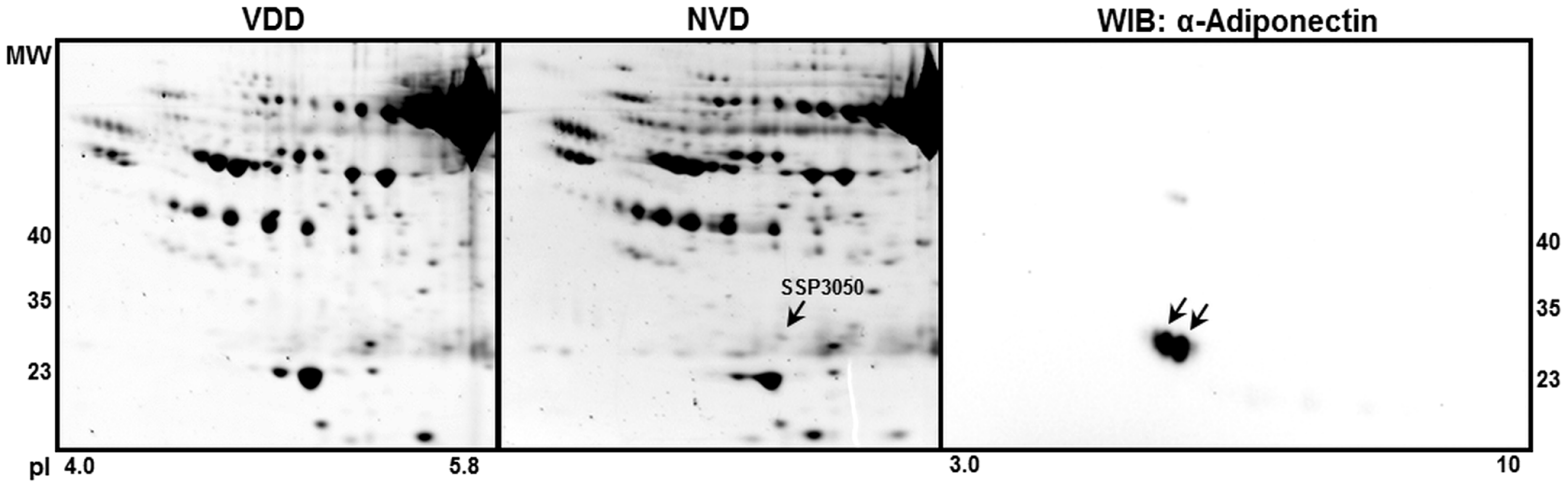
Proteomic evaluation predicts adiponectin isoforms as being differentially expressed between VDD and NVD. A 2D-electrophoretic analysis was performed in duplicate for the 42 subjects using IPG3-10, with proteins detected by Sypro Ruby staining. Spot/s predicted to be adiponectin are indicated by the PDQuest identification number (SSP3050). Supportive evidence for the prediction was given by performing a WIB of 2D-electrohoretic gels using anti-adiponectin antibody. Representative gels are shown.

Plasma from both VDD and NVD subjects were analyzed by 2D-electrophoresis blinded to evaluate differences in the expression and post-translational modifications (PTM) of circulating proteins. A global analysis using IPG 3-10 in duplicate for each subject, identified, when corrected for Sypro-Ruby background anomalies, 53 “spots” that were significantly different between the two groups (p<0.05), with the top ten most significant spots identified by PDQuest shown (Table 2). Of the 53 spots, 51% were downregulated. Amongst the spots predicted to be downregulated between VDD and NVD, was the adipokine adiponectin (ID 3050) identified using Swiss-Prot human plasma database analysis in combination with Tagldent Searches (http://web.expasy.org) with pI and MW PDQuest estimates (Figure 1). A WIB analysis of 2D-electrophoretic gels using an adiponectin specific antibody, identified 2 adiponectin monomeric isoforms within the pI5.4/30kDa range, providing further evidence that the original “spot” could be adiponectin and a PTM of adiponectin (Figure 1).

**Table 2:** Top ten most significantly modulated plasma proteins between VDD and NVD obese pediatric subjects.

PDQuest ID	VDD Media n = 18 (AU)	NVD Media n = 24 (AU)	*P*-value VVD vs NVD	MW kDa*	pI*	Protein ID**	Accession No.
302	35.1	75.5	*0.007*	54	4.8	N/D***	-
601	28.9	12.7	*0.008*	100	3.2	N/D	-
1101	30.2	20.9	*0.01*	39	4.8	N/D	-
1103	278	348.8	*0.04*	40	5.2	Haptoglobin β	P00738
1402	128.2	94.5	*0.04*	62	4.8	N/D	-
2301	278.2	216.2	*0.04*	55	5.0	N/D***	-
3003	84.1	63.5	*0.006*	26	5.5	N/D	-
**3050**	**22.8**	**31.3**	***0.03***	**28**	**5.4**	**Adiponectin**	**Q15848**
6603	18.6	60.3	*0.02*	100	6.4	N/D	-
7305	53.7	42.8	*0.02*	55	6.3	N/D	-

PDQuest estimate; ** Confirmed by WIB; N/D = not determined; *** Under investigation.

### Adiponectin confirmational evaluation

**Figure 2:**
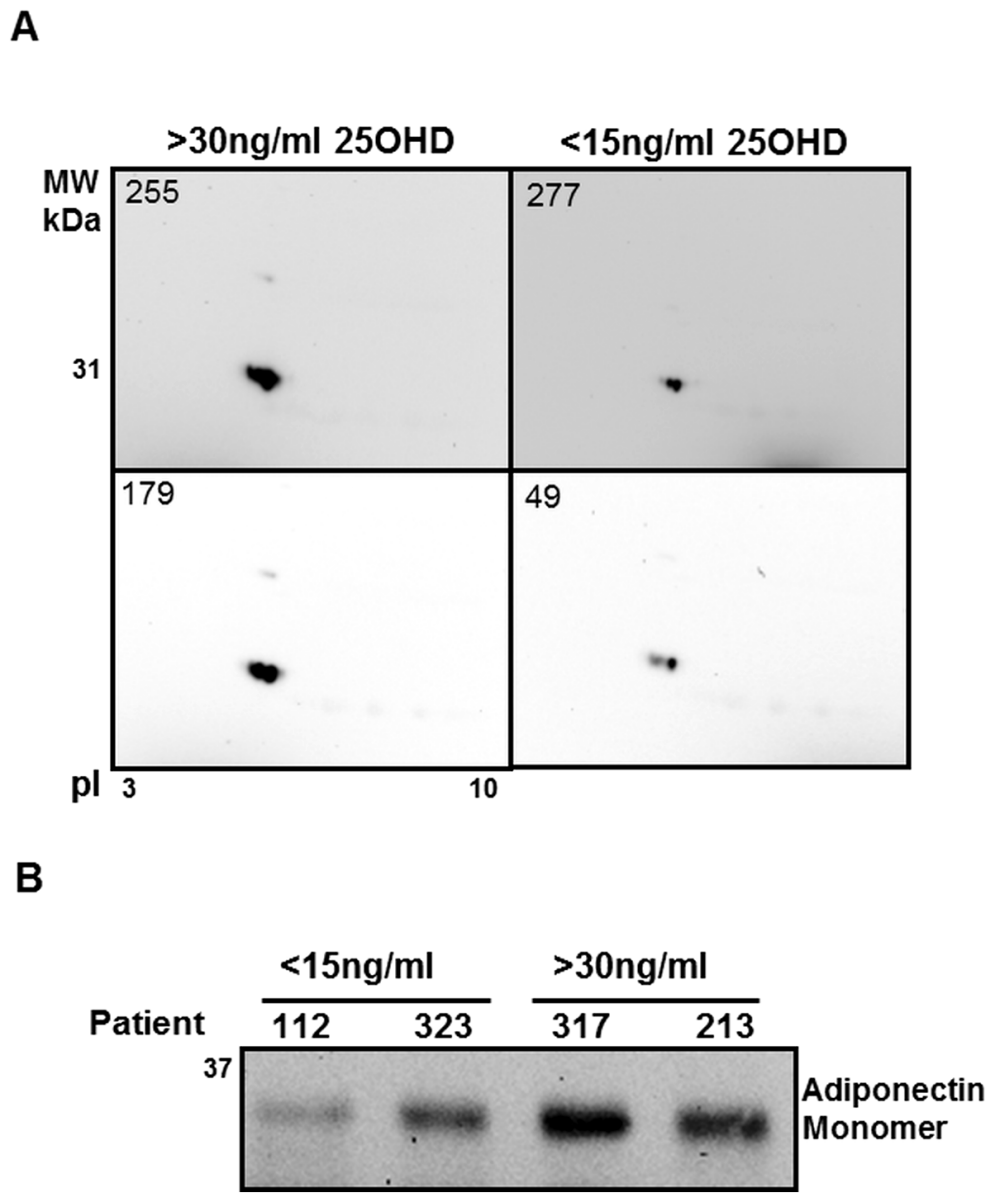
WIB of proteomic analyses and plasma samples confirms that total adiponectin is decreased in VD deficient pediatric obese subjects (VDD) when compared to pediatric obese with normal VD levels (NVD). **A.** A WIB of 2D-electrophoretic analyses was performed in VDD (n = 10) and NVD (n = 10) subjects using anti-adiponectin antibody. **B.** A WIB analysis under reduced conditions of total adiponectin in the plasma of representative VDD (<15 ng/ml) and NVD (>30 ng/ml) subjects.

Adiponectin was selected for confirmational studies due to its strong correlation with obesity and its co-morbidities, as well as its localization within one of the susceptibility gene loci for obesity [25]. Two approaches were utilized to confirm the differences in plasma adiponectin expression between VDD and NVD obese pediatric subjects. In the first, WIB analyses of 2D-electrophoretic gels were performed in 10 subjects from each group, with this approach demonstrating qualitatively reduced total adiponectin in subjects with <15 ng/ml 25-OHD_3_ (VDD; Figure 2A). In the second approach, densitometric WIB evaluations (VDD vs NVD; 7187±383 vs 8594±587 AU; p<0.035; Figure 2B) and ELISA evaluations (VDD vs NVD; 6.0±0.8 vs 10.9±1.9 µg/ml; p<0.05) of circulating total adiponectin in all subjects, further confirmed that total adiponectin is reduced in pediatric obese subjects with <15 ng/ml 25-OHD_3_ with no alteration in the significance when corrected for BMI-SDS, age and sex.

**Figure 3:**
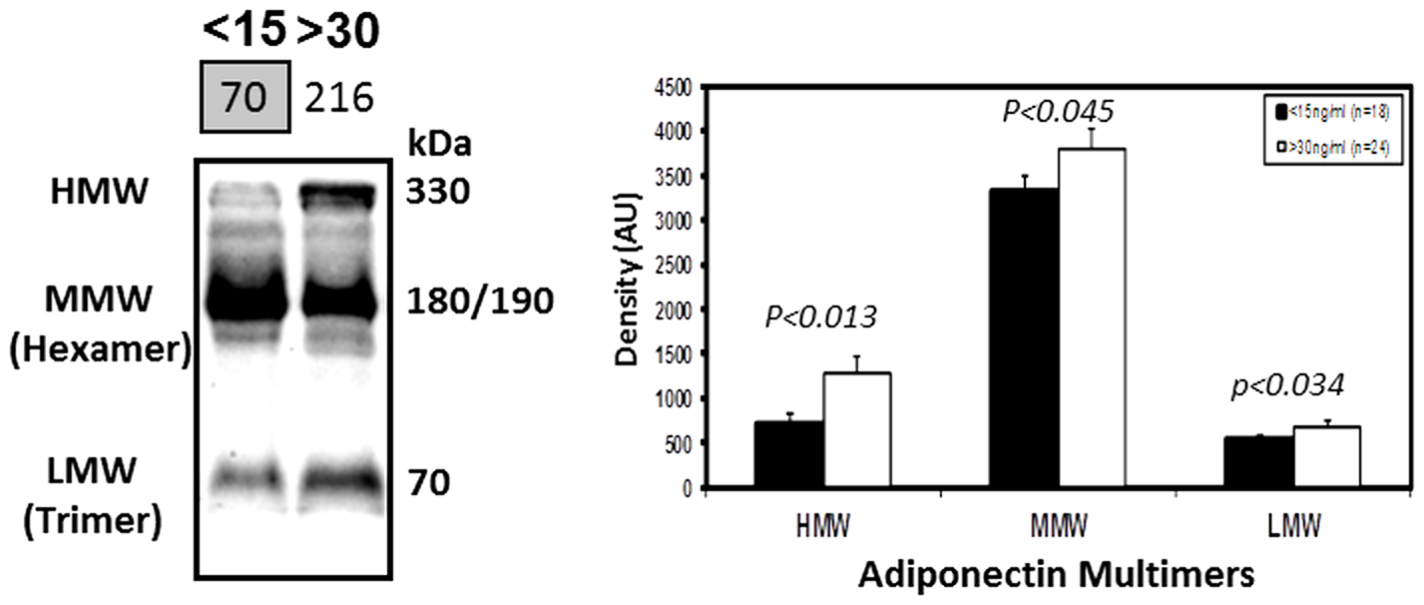
The multimeric forms of adiponectin are reduced, in particular the HMW form, in VD deficient pediatric obese subjects. A WIB analysis under non-reduced conditions and a quantitative densitometric analysis of the multimeric forms of adiponectin (HMW, MMW, LMW) in the plasma of representative VDD (<15 ng/ml; n = 18) and NVD (>30 ng/ml; n = 24) subjects. Densitometric results were normalized to plasma protein concentrations.

Adiponectin circulates in plasma in three major forms: trimers/low molecular weight (LMW), hexamers/medium-MW (MMW) and high-MW (HMW), with the HMW form shown to be the most bioactive, particularly with respects to insulin action [25]. To dissect changes in the adiponectin isoforms, an evaluation using non-reduced (NR)-WIB of plasma from both VDD and NVD subjects was performed. While showing that HMW, MMW and LMW forms were all significantly lower in VDD subjects, the greatest difference was observed for HMW adiponectin (VDD vs NVD; 697.1±127.7 vs 1270.5±198 AU; p<0.013; Figure 3).

### VD-treatment: Clinical and adiponectin evaluations

To understand the benefits of VD3 therapy, the original 18 VDD were administered 3000 IU cholecalciferol (VD3) once a week, for a 12mo study. Of the 18 subjects, 10 concluded the study, while the remainders discontinued the treatment with cholecalciferol or dropped out with clinical controls. In the 10 VDD subjects, 25-OHD3 levels increased during the course of the treatment (10.6±0.6 vs 20.4±4.8 ng/ml; p<0.04), while DBP (84.2±4.6 vs 77.5±3.2 mmHg) and fasting glucose (88.0±3.1 vs 81.5±3.1 mg/dl) decreased (p<0.05) without significant changes in other parameters or BMI (26.1±1.7 vs 26.3±2.1 Kg/m^2^) at 12 months. When corrected for BMISDS, age and sex, the significance for fasting glucose was lost. To overcome the absence of a direct measurement of fat tissue, we measured visceral adiposity index (VAI), an indicator of visceral adiposity status and function [26], body adiposity index (BAI), which correlates more than BMI with the percentage of body fat measured by dual energy X-ray absorptiometry [27] and ghrelin which increases in case of adiposity loss [28]. Waist circumference (90.2±3.9 vs 91.3±5.3 pg/ml), waist-to-height ratio (0.63±0.02 vs 0.63±0.05), VAI (1.893±0.337 vs 1.852±0.343), BAI (0.056±0.001 vs 0.058±0.002 Kg/m^2^), and ghrelin (106.0±21.1 vs 98.1±30.6 pg/ml) did not change from baseline to T12 suggesting that fat mass did not decrease over time.

**Figure 4:**
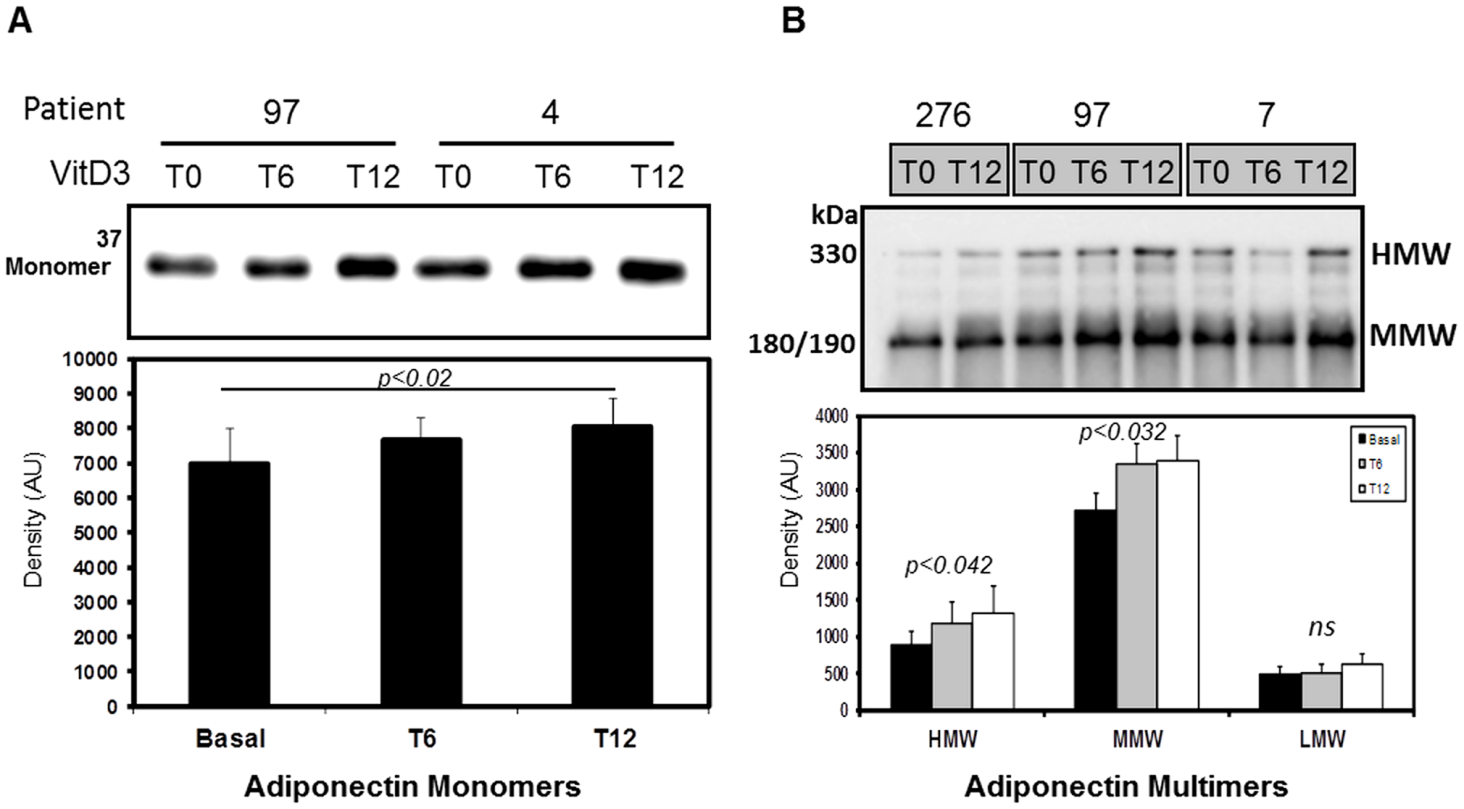
Total and the HMW and MMW multimeric forms of adiponectin increase in VD deficient pediatric obese subjects following cholecalciferol supplementation for 12mo. **A.** A WIB analysis under reduced conditions and a quantitative densitometric analysis of total adiponectin in the plasma of representative VDD (<15 ng/ml) and NVD (>30 ng/ml) subjects. **B.** A WIB analysis under non-reduced conditions and a quantitative densitometric analysis of the multimeric forms of adiponectin in the plasma of VDD (<15 ng/ml; n = 18) and NVD (>30 ng/ml; n = 24) subjects.

With respects to adiponectin levels, a gradual and modest, yet significant improvement in total adiponectin was observed over the 12mo period, as demonstrated by WIB (Figure 4A). Similar results were also observed for the multimeric forms of adiponectin in circulation. The NR-WIB analysis demonstrated that both the HMW and MMW forms improved modestly yet significantly with cholecalciferol therapy, while alterations in LMW adiponectin did not reach significance (Figure 4B). Correction for covariates did not modify the results.

### Vitamin D3 treatment upregulates adiponectin and disulfide bond-A oxidoreductase-like protein (DsbA-L) promoting adiponectin multimerization in 3T3-L1 mature adipocytes

**Figure 5:**
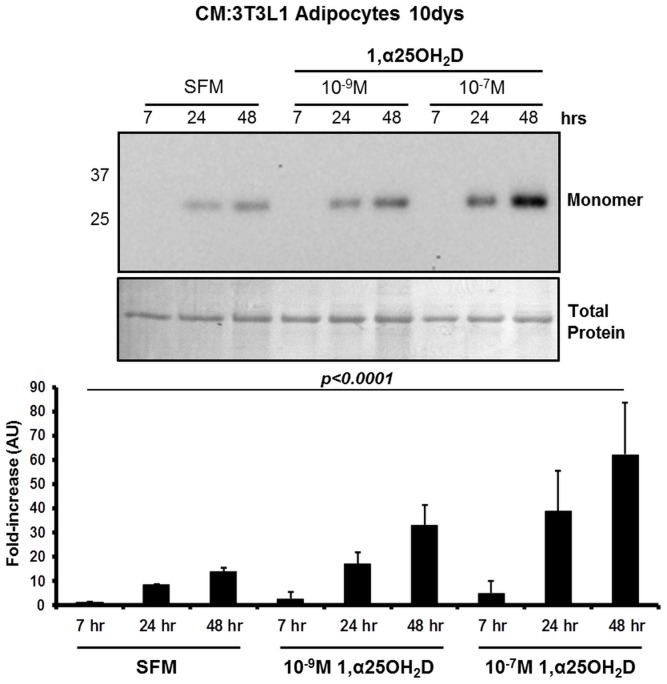
Total adiponectin secretion increases in 3T3-L1 adipocytes treated with 1α,25-(OH)2D3. 3T3-L1 adipocytes, generated using a standard differentiation protocol, at day 10 were treated with increasing concentrations of 1α,25-(OH)2D3 (10^−9^ to 10^−7^ M) in SFM or SFM with vehicle for 48 h. The CM at 7, 24 and 48 h from the same treatment was analyzed by WIB under reduced condition and analyzed densitometrically for the synthesis of adiponectin. Results were normalized to α-tubulin and are presented as fold-increase with respect to the 7 h SFM sample (n = 4).

**Figure 6:**
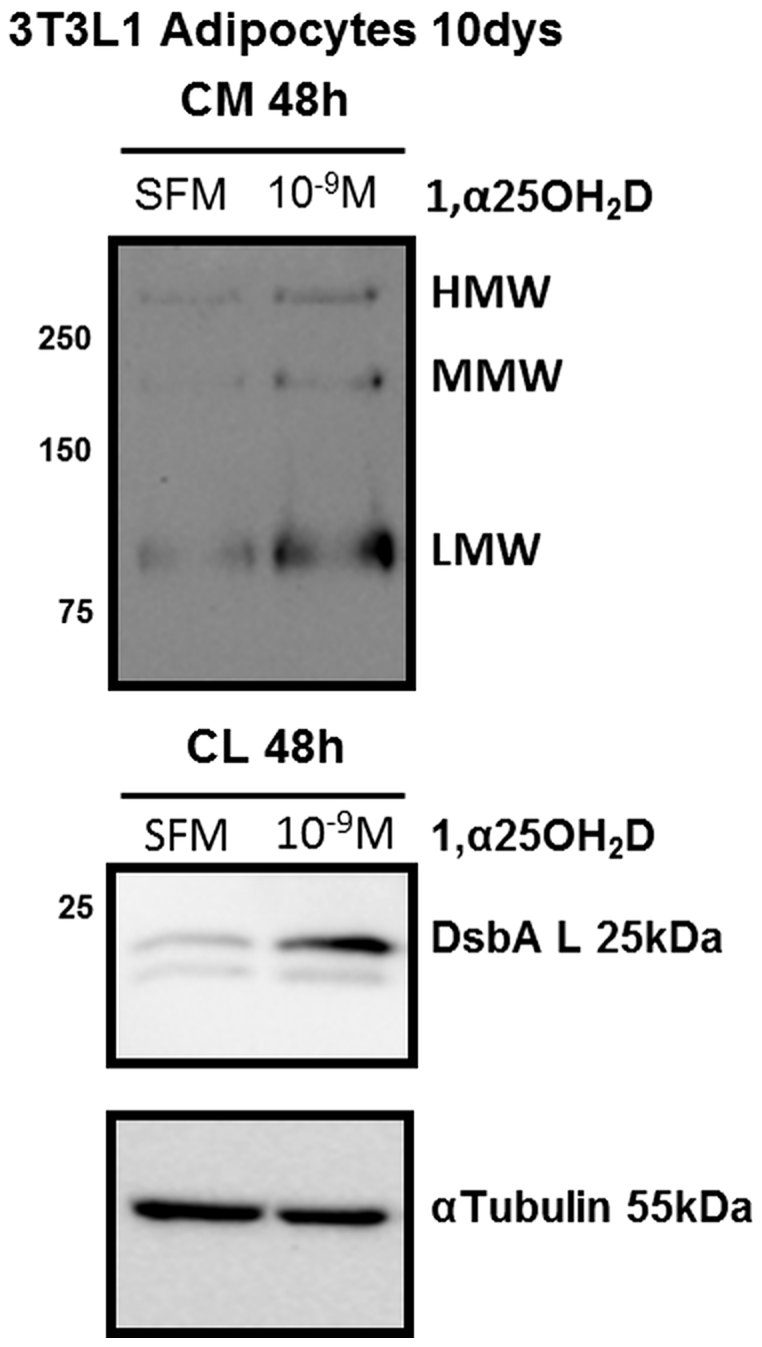
The secretion of the adiponectin multimeric forms, in particular the LMW form, in parallel with the ER-chaperon DsbA-L, increases in 3T3-L1 adipocytes treated with 1α,25-(OH)2D3. 3T3-L1 adipocytes, generated using a standard differentiation protocol, at day 10 were treated with increasing concentrations of 1α,25-(OH)2D3 (10-9 to 10-7M) in SFM or SFM with vehicle for 48 h. The CM at 7, 24 and 48 h from the same treatment and CL at 48 h, was analyzed by WIB under non-reduced or reduced conditions for the synthesis of adiponectin multimeric forms or DsbA-L with α-tubulin, respectively.

To examine the direct effect of VD on adipocytes, the cellular component of AT exclusively responsible for the production and secretion of adiponectin, 3T3-L1 cells were induced to differentiate into adipocytes. Adipocytes were then treated for a further 48 hr period in SFM with or w/o increasing concentrations (10^−9^ – 10^−7^ M) of the bioactive form of VD3, 1α,25-(OH)2D3, with aliquots of CM removed at 7, 24 and 48 hr. A WIB analysis of monomeric adiponectin in the CM, demonstrated a significant increase in total adiponectin secretion with increasing concentrations of 1α,25-(OH)2D3 and with respect to time (n = 4; Figure 5). An evaluation of the secretory capacity of the cells by examining total protein within the CM, demonstrated that secretory profile is unchanged and as such the upregulation of adiponectin secretion/production by 1α,25-(OH)2D3 is selective. We also observed with the higher concentration of 1α,25-(OH)2D3, while adiponectin accumulation in the CM continued, there was a deterioration in cell quality and α-tubulin expression, as such the lower concentration was selected for further investigations (data not shown), with the most significant changes evident for LMW form which increased 8-fold (p<0.01) over the time period (Figure 6). An analysis of the adiponectin interactive protein, DsbA-L, which has been demonstrated to promote adiponectin multimerization in adipocytes [29], [30], demonstrated a higher expression level in 3T3-L1 adipocyte cell lysates treated with 10^−9^ M 1α,25-(OH)2D3 for 48 hr with respect to SFM, supporting the increased multimerization of adiponectin following 1α,25-(OH)2D3 treatments (Figure 6). It is important to note that while there was an overall increase in adiponectin multimerization in treated 3T3-L1 cells, there was a clear predominance of the LMW form in both the basal and treated state in clear contrast to the profile present in human plasma samples.

## Discussion

Pediatric obesity is an emerging health issue in many developed countries, with repercussions later in life. Like adult obesity, it is conceivable that genotype, lifestyle and behavioral factors such as energy intake together with the levels of physical activity, play critical roles in the obesity epidemic. There is, however, now evidence suggesting that VD may contribute to the regulation of weight gain, particularly in association to energy-restricted diets [3]. As such, VD status has been proposed as a promising strategy for the prevention of obesity and the development of its associated complications. To date though, results of clinical studies regarding the role of VD in obesity are inconclusive, with the “concert” of metabolic signaling pathways that link obesity with VD status, undefined [15]. In the present study, we used a proteomic approach to identify potential *in vivo* biomarkers that could provide a link between VD deficiency and pediatric obesity. Using this technology, the multimeric forms of adiponectin, in particular the HMW form, were identified as being downregulated in obese pediatric subjects with vitamin D deficiency which in turn could be upregulated with VD3 supplementation, independently of BMI. Further, a direct effect of 1α,25-(OH)2D3 on adipocytes was demonstrated, with adiponectin and its multimeric forms, as well as the adiponectin interactive protein, DsbA-L, upregulated by 1α,25-(OH)2D3 treatment at low pharmacological concentrations.

The cohort of children enrolled in the present study who were VD deficient, were more obese, more insulin-resistant and had higher fasting glucose and DBP. These data are in agreement with those found in a larger population covering the pediatric age [10], [31]. In particular, higher fasting glucose and blood pressure levels observed in our cohort are in line with data of 2001–2004 National Health and Nutrition Examination Survey in US adolescents [10]. VD deficiency is common in obese patients and it is possible that this is the result of many factors, such as a decreased VD bioavailability due to sequestration in adipose tissue [32], low dietary VD intake due to poor nutritional habits and minimal sun exposure due to a sedentary indoor lifestyle [33]. Although it is known that morbid obesity is directly correlated with higher insulin resistance, fasting glucose levels and other comorbidities such as hypertension, VD deficiency is associated with numerous biomarkers of systemic inflammation and metabolic impairment, regardless of the total fat mass [10]. Moreover, the 15 ng/ml or less of VD may be the threshold by which VD deficiency confers negative effects on insulin sensitivity [10], [34] and also hazard ratio for cardiovascular events [17]. We showed that 1 year cholecalciferol treatment improved DPB and fasting glucose without significant changes in terms of BMI. Although in a small group of children, these data are in line with other observations in some pilot studies in animals [35] and in adults [36]. As our patients did not improve their weight, our results seem dependent on VD without an influence of body fat changes. It has to be underlined that our children increased VD levels at the threshold of deficiency for bone effects [15], suggesting that pleiotropic actions other than those on bone would be exerted at different levels as suggested by cross sectional studies which indicate 15 ng/ml as the cut off for the cardiovascular disease risk [10], [34].

To explore the “concert” of metabolic signaling pathways which underlie the link between VD deficiency and obesity, a 2D-based proteomic methodology investigated the global changes in expression levels as well as PTMs associated with VD status. In our cohort of pediatric obese subjects divided according to their circulating levels of 25-OHD3, adiponectin was identified and confirmed to be significantly decreased in 25-OHD3 deficient obese pediatric subjects. Adiponectin has been demonstrated to have insulin-sensitizing effects [37], regulates centrally food intake and body weight [38] and possesses cardioprotective [39], anti-inflammatory and anti-oxidant properties [40], demonstrating that it has a clear clinical relevance with respect to obesity and its associated complications. Adiponectin is abundantly produced by adipose tissue with its synthesis and secretion specific to adipocytes [41] In contrast to other adipokines, circulating adiponectin is negatively correlated with BMI and is decreased further in patients with insulin resistance, type 2 diabetes and cardiovascular disease [37]. In the present study, we observed within the pediatric obese population a further subdivision in circulating total adiponectin levels according to VD levels, with VD deficient subjects presenting significantly lower levels of circulating adiponectin.

Within the circulation, adiponectin is present in three multimeric forms: trimer (LMW), hexamer (MMW) and HMW (12–18 monomers), with the HMW form considered to be the key bioactive form, particularly with respect to insulin action [25], [37], [42]. In the present study, we demonstrated that all molecular weight forms were decreased in those with VD deficiency with all analyses performed. Circulating concentrations of adiponectin are known to be significantly decreased with the development of obesity and with altered glycemic control with the HMW form more strictly involved in insulin resistance [25], [37], [42]. From a clinical perspective it can be hypothesized that total adiponectin and its multimeric forms were reduced in VD deficient children because they are more obese. However, in the present study we have shown an increase in circulating adiponectin levels, in particular of HMW form, after 1 yr cholecalciferol treatment. This significant improvement occurred regardless of weight reduction, suggesting a direct role of VD. The significantly higher levels of adiponectin could be one of the key factors which contribute to the shown improvement of their metabolic phenotype, as previously demonstrated by numerous studies (for review see 36), including in children where an increase in HMW adiponectin was shown to be correlated with an improvement in insulin sensitivity [43], [44].

With the *in vivo* data supporting a direct role for VD in the regulation of adiponectin, and to confirm that VD yields an effect on adiponectin expression, the direct effect of VD on adiponectin secretion was tested using the murine 3T3-L1 adipocyte cell model. It is known that nuclear and membrane VDR have been demonstrated in a large array of tissues, including adipose tissue [45] and is expressed in 3T3-L1 cells, with the highest expression observed during the early stages of adipocyte differentiation where VD has been shown to inhibit the differentiation process [46], [47]. In the present investigation, we conferred with the clinical observations in VD deficient pediatric obese subjects, with adiponectin secretion and the multimeric forms increasing in 3T3-L1 mature adipocytes following 1α,25-(OH)2D3 supplementation, with significant effects observed at very low pharmacological concentrations.

Previous studies regarding the direct effects of 1α,25-(OH)2D3 on adipose tissue are controversial with both inflammatory and anti-inflammatory effects being reported [48]–[50]. Lorente-Cebrián *et al.*, [47] reported a downregulation in the secretion of total adiponectin in human sub-cutaneous-AT (SAT) primary culture differentiated adipocytes treated with 1α,25-(OH)2D3, with no effect on mRNA expression. They also observed a downregulation in the pro-inflammatory marker monocyte chemoattractant protein-1 (MCP-1), supporting the concept of dual roles for 1α,25-(OH)2D3 in adipose tissue inflammation. A downregulation of key pro-inflammatory markers by 1α,25-(OH)2D3 was also demonstrated by Gao *et al.*, [50] specifically in preadipocytes, suggesting that the preadipocyte population is the major source of proinflammatory mediators. A possible explanation for the divergent results is that these studies observed their effects using 10-100-fold higher concentrations of 1α,25-(OH)2D3 than that used in the present investigation, where we observed that such concentrations had deleterious effects on adipocytes, most likely through the activation of the Ca^2+^-mediated apoptotic pathway [12]. Other differences between the study by Lorente-Cebrián *et al.*, [49] and the present investigation, is the use of adult human female primary culture SAT adipocytes versus murine 3T3-L1 adipocytes. Further, our study was not directed solely to total adiponectin secretion, but it also examined the multimeric adiponectin secretory profile following 1α,25-(OH)2D3. Here we observed an altered distribution in the CM of these cells when compared to a human plasma profile, with a clear predominance of the LMW form, suggesting clear species diversity with respect to the synthesis and secretion of adiponectin. Our experiments were also performed in SF conditions suggesting in addition to species diversity, they may also be a dependence on other serum activators in the regulation of adiponectin which were absent in our study. Overall, while our *in vitro* data support our clinical findings, it would be of relevance to approach our *in vivo* findings using human adipocytes addressing at the same time the depot specific differences in adiponectin secretion as previously described [51], excluding also the sex related differences as well as those likely present between adults and children.

While in the present study it can’t be excluded that there is a direct effect of 1α,25-(OH)2D3 on 3T3-L1 adipocyte transcription, it is feasible that the increased synthesis and multimerization of adiponectin is dependent on the induction of endoplasmic reticulum (ER) genes involved in the post-translational process of multimerization. In fact, we observed in response to low concentrations of 1α,25-(OH)2D3, an increase in the ER-chaperon DsbA-L protein, a key regulator of adiponectin folding and assembly [52], which paralleled the increase in adiponectin synthesis and multimerization. The expression levels of DsbA-L are regulated in response to ER-stress and have been shown to be significantly reduced in obese subjects and mice [29], [30]. While we observed that the ratio of LMW to the MMW and HMW were diverse to the human plasmatic profiles, most likely a result of species diversity or the absence of a key regulatory protein/s in the SF CM, the results are in concordance with other studies using thiazolidinediones [53]. Taken together, these results demonstrate that increase in adiponectin levels and multimerization by 1α,25-(OH)2D3, may occur via post-transcription-dependent mechanisms involving ER proteins, such as DsbA-L.

There are limitations in the present study. The first is the absence of true body fat measurements through radiological techniques. It can be speculated that adiponectin changes are due to changes in fat mass, however our subjects did not improve their weight in terms of BMI, which is a good surrogate measurement for body fat in obesity [54]. Moreover, BMISDS, waist, waist-to-height-ratio, BAI, VAI and ghrelin levels did not change, suggesting that fat mass did not decrease. Further, it has been demonstrated that the impairment of total and HMW-adiponectin levels in childhood obesity is different to adult obese patients, showing less of a relationship with body fat content [44]. Similarly, recent placebo controlled studies in humans observed an increase in adiponectin during VD supplementation [55], [56]. Furthermore, BAI which has been demonstrated to be a good indirect marker of body fat percentage and is superior to BMI [27], was unchanged, supporting the hypothesis that adiponectin is modulated by VD. Similarly, VAI, which has been proposed to be an indirect marker of visceral adipose dysfunction [26], remained unchanged. It has been demonstrated that with the increase of VAI, adiponectin progressively decreases [57]. The fact that VAI remained unchanged and adiponectin increased in our population treated with VD, provides further evidence of a direct role of VD on adiponectin production. This is also supported by the demonstration of stable ghrelin levels, which are a precocious index of the recovery of an ideal body weight when they increase. The second limitation is we performed just two adiponectin evaluations after VD treatment, with more prolonged studies and more frequent time points needed to surely prove a connection. Despite this, when we weighted adiponectin for cofounders, which may influence its secretion, the significance was maintained suggesting an influence of the VD treatment. Thirdly, this is a pilot study to investigate whether proteins identified via a proteomic approach are directly modulated by VD, in the case of adiponectin, an *in vitro* adipose tissue model. Notably, more studies are needed in the future to understand the biological mechanisms and whether other proteins are implicated. In support of the findings however, a diet rich in VD has been shown to increase adiponectin synthesis in swine epicardial adipose tissue [58].

VD has been proposed as a promising strategy for the prevention of obesity and the development of its associated complications. While VD has a long history, the “concert” of metabolic signaling pathways that link obesity with VD status remain undefined. In the present study, we used a proteomic approach to study the global plasmatic changes between VD deficient and normal obese pediatric subjects identifying that the multimeric forms of adiponectin, in particular the HMW form are plasmatic biomarkers that could provide a mechanistic link between VD deficiency and pediatric obesity, with total plasma levels increasing with cholecalciferol supplementation. By using the *in vitro* 3T3-L1 adipocyte cell model system, a direct effect of 1α,25-(OH)2D3 at low pharmacological concentrations was demonstrated. While the mechanism of VD control over adiponectin remains to be clearly defined, the upregulation of the ER-chaperon DsbA-L, suggests that this may be a post-transcriptional dependent event.
